# Spelthorne Sanatorium

**Published:** 1890-07-19

**Authors:** 


### SPELTHORNE SANATORIUM.

SPELTHORNE SANATORIUM is situated just one [illegible] from the Feltham station of the London and South-Western Railway, and is quietly doing a useful work in the reformation of women who have fallen into habits of intemperance. Ladies and middle-class, and working women are admissable, but themselves or their friends are obliged to make some provision towards their maintenance; and prison, penitentiary, or workhouse cases are ineligible. Those who carry on the institution give their services voluntarily, so that the payments mentioned do not represent profits, but are simply contributions which are added to voluntary subscriptions in order to provide the necessary income. The Wantage sisters are in charge of the sanatorium, and the chaplains, the secretaries, the medical adviser, and the treasurer give their time and work gratuitously. Amongst the patients, women who give assistance in the laundry or the sewing room thereby greatly reduce their payments for maintenance, and we are glad to see a substantial profit entered to the credit of the laundry, and hope that the appeal for additional "sewing-work" will be successful. Employment is, in fact, an important part of the "treatment" pursued here.

The sanatorium has been eleven years in existence, and can receive nearly 20 patients. With more house accommodation nearly double the number of patients could be received, and this with very trifling additional expense for workers. There were as many as 88 applications for admission last year, and the committee make a reasonable appeal for help to enable them to extend the benefits of the institution. Some 300 subscribers of small sums provide about £200 a-year; patients' payments come to £520 18s. From the laundry and the needlework, some additional revenue is derived. On the other side, the expenses for maintenance, rent, wages, medicines, &c., are very considerable; but the sisters (with the help of the committee), or the committee (with the help of the sisters), manage everything so admirable that the sanatorium stands clear of debt.

Five pounds is the highest sum we see in the list of subscriptions and donations, and sums of £1, of 10s., and of 5s. are of very frequent recurrence. A few large gifts from wealthy people, especially now that funds are sought for enlarging the building, would be well bestowed, and would not, we believe, diminish the interest in this undertaking by the present very creditable number of subscribers of small sums. An institution which annually sends back to husbands and children women who have been cured of intemperate habits, is one which has strong claims upon the liberality of the public. Major-General Fooks (5, Barrett Road, W.) would gladly give full particulars to persons disposed to assist in this practical and philanthropic enterprise.

